# Evaluation of Winter Footwear: Comparison of Test Methods to Determine Footwear Slip Resistance on Ice Surfaces

**DOI:** 10.3390/ijerph18020405

**Published:** 2021-01-06

**Authors:** Atena Roshan Fekr, Yue Li, Chantal Gauvin, Gordon Wong, Wayne Cheng, Geoff Fernie, Tilak Dutta

**Affiliations:** 1 The Kite Research Institute, Toronto Rehabilitation Institute—University Health Network, Toronto, ON M5G 2A2, Canada; yue.li@uhn.ca (Y.L.); gordon.wong@uhn.ca (G.W.); wayne.cheng@uhn.ca (W.C.); Geoff.Fernie@uhn.ca (G.F.); tilak.dutta@uhn.ca (T.D.); 2 Institute of Biomedical Engineering, University of Toronto, Toronto, ON M5S 3G9, Canada; 3 Mechanical and Physical Risk Prevention Team, IRSST—Institut de Recherche Robert-Sauvé en Santé et en Sécurité du Travail, Montréal, QC H3A 3C2, Canada; chantal.gauvin@irsst.qc.ca

**Keywords:** slip, fall, slip resistance, footwear, maximum achievable angle, SATRA, winter, ice

## Abstract

The use of slip-resistant winter footwear is crucial for the prevention of slips and falls on ice and snow. The main objective of this paper is to evaluate a mechanical testing method to determine footwear slip resistance on wet and dry ice surfaces and to compare it with the human-centred test method introduced by researchers at KITE (Knowledge, Innovation, Talent, Everywhere)-Toronto Rehabilitation Institute-University Health Network. Phase 1 of this study assessed the repeatability and reproducibility of the mechanical method by evaluating ten different occupational winter boots using two SATRA Slip resistance testers (STM 603, SATRA Technology Centre, Kettering, UK). One tester is located in Toronto and one in Montreal. These boots were chosen based on the needs of the IRSST (Institut de Recherche Robert-Sauvé en Santé et en Sécurité du Travail, Montréal, Quebec, Canada), who were primarily interested in providing safe winter footwear for police, firefighters and municipal workers. In Phase 2, the results of the human-centred test approach were compared with the mechanical results. In Phase 3, two of these boots with conflicting results from the previous phases were tested using a second human-centred method. In Phase 1, the mechanical testing results obtained in the two labs showed a high linear correlation (>0.94) and good agreement on both ice surfaces; however, they revealed a bias (~0.06) between the two labs on the dry ice condition. The mechanical and human-centred tests (phase 2) were found to be better correlated in the wet ice condition (R = 0.95) compared to the dry ice condition (R = 0.34). Finally, the rating of the footwear slip resistance based on the number of slips counted in phase 3 was consistent with the rating by the human-centred test method (phase 2), but not the mechanical method (phase 1). The findings of this study provide a better understanding of the limitations of the SATRA ice tray for measuring footwear slip resistance and demonstrate that the mechanical method must be further refined to make it more comparable to the human-centred methods to achieve better agreement with real-world performance.

## 1. Introduction

Slips and falls on ice and snow are widespread in many winter-experiencing countries [1,2,3,4,5]. In Canada, statistics from various parts of the country reveal how extensive slip and fall accidents are. For example, about 30,000 emergency department visits and 2800 hospitalizations from falls on ice and snow were documented in 2006–2015 in Toronto, one of Canada’s largest cities [6,7]. Similarly, recent reports between April 2017 and March 2018 show that 1673 hospitalizations were observed in the province of Ontario due to falls on ice, contributing to the national total of about 8500 hospitalizations [8]. In the province of Quebec, the number of hospitalizations from fall-related winter injuries was about 2500 from April 2017 to March 2018 [8]. In the cities of Laval and Montréal Island, approximately 960 outdoor falls required ambulance use over two months of December 2008–January 2009 [9]. Six hundred twenty-five of these 960 falls (65%) were specifically attributed to falls on ice and snow. Interestingly, these numbers support the documented association between an increased rate of falls in older adults living in Montréal with weather warnings of freezing rain/icy conditions [10]. Injuries related to falls also place a significant economic burden on Canada. Estimates suggest that the costs of fall-related emergency visits in older adults are about $11,000, with hospitalizations costing on average $29,000 USD [11].

The challenge during gait is that the body’s centre of mass (COM) is continually thrown ahead of its base of support and safe placement of the swinging foot is critical for preventing a loss of balance at each step [12]. Furthermore, the body acts as an inverted pendulum, which is inherently unstable due to the forward momentum of the head, arms, and trunk while walking, since the location of the COM is approximately two-thirds of body height above the ground [12]. The major risk factor for a slip is low friction between footwear and the walking surface [2,4,13,14]. Thus, slips generally occur at heel contact and toe-off where the shear forces are the highest and the COM passes outside the base of support [12,15]. A slip occurring at heel contact is called a heel slip while a slip occurring at toe-off is referred to as a toe slip [15]. Heel slips result in a forward slip with the leading foot, which is likely to cause a backwards fall since the forward momentum of the body keeps body weight on the slipping foot [16]. In contrast, toe slips result in a backward slip on the trailing foot, which may result in a forward fall [15].

However, there are very few available methods for assessing the slip resistance of footwear on icy surfaces. [17,18]. A human-centred test method has been developed by researchers at KITE (Knowledge, Innovation, Talent, Everywhere)Toronto Rehabilitation Institute, University Health Network (KITE) to evaluate footwear slip resistance on ice surfaces [19,20,21,22]. Tests are done inside the WinterLab, which is one of KITE’s Challenging Environment Assessment Laboratories (CEAL). WinterLab has a real ice floor that can reach sub-zero temperatures. The method, called the Maximum Achievable Angle (MAA) test, measures the steepest incline that participants can walk up and down while wearing test footwear without experiencing a two-foot slip [21,22]. The method can be performed on melting wet ice and smooth cold dry ice [23]. The equivalent coefficient of friction (COF) values are obtained by taking the tangent of the incline angles, as in the calculation for a ramp test [24,25]. The MAA test method has demonstrated good repeatability and consistent results when ranking footwear on various icy surfaces [19,20,26]. This method has also shown to be a useful and valid test in a study, where winter boots with high MAA scores worn by workers have actually been involved in fewer slips than those in a control group [22,27].

A shoe’s slip resistance can also be assessed mechanically by measuring the COF between the shoe outsole and a test surface [28,29]. There are several mechanical devices to measure the COF [30,31]. The STM 603 Slip resistance tester, developed by SATRA Technology Centre (Kettering, UK) [32,33], is one of the most widely used mechanical devices for COF measurement by standard organizations and the footwear industry. The proprietary SATRA test method [34] provides guidelines to test footwear on ice surfaces and describes different types of ice surfaces that can be created using a refrigerated ice tray (STM 603ICE from SATRA Technology Centre, Kettering, UK), including frosted ice, smooth dry ice and wet ice. The STM 603 Slip resistance tester can perform standard mechanical testing (ASTM F2913-ASTM international, West Conshohocken, PA, USA or EN ISO 13287—ISO, Geneva, Switzerland) on ice surfaces. However, little information has been published about the standard mechanical test method using the refrigerated ice tray, while the repeatability and reproducibility of such tests on ice surfaces have not been assessed.

Some studies have evaluated the ability of the standard mechanical test method to provide a footwear slipperiness measurement representative of the actual footwear performance experienced by human participants walking on contaminated indoor surfaces [35,36,37]. Human-based approaches are more externally valid means of assessing footwear function, since they take into account the capacity of human beings to adapt their gait to hazardous conditions [15,38]. Therefore, it is essential to establish the accuracy of the mechanical method to test the whole shoe on ice surfaces.

The goals of this study were to explore and compare existing evaluation methods for slip resistance on ice and to consider new measurement and analysis methods that can help to demonstrate which methods can best reflect real-world performance. The study was conducted in three phases with the following objectives:

Phase 1:

(A) Develop an alternative ice surface preparation protocol for the mechanical method based on the existing SATRA TM144 protocol. In order to compare the mechanical method with the MAA, the ice surface conditions in these two methods had to be comparable.
(B) Evaluate the repeatability and reproducibility of the mechanical method for measuring footwear slip resistance on the ice surfaces at two different laboratories: KITE and Institut de Recherche Robert-Sauvé en Santé et en Sécurité du Travail in Montréal (IRSST).

Phase 2:

Compare the results from the mechanical method with the MAA human-centred method for the evaluation of footwear slip-resistance performance on ice surfaces, using two methods for calculating the MAA score to determine which method is most appropriate for consumers.

Phase 3:

In the case of inconsistency between the results of mechanical and human-centred methods, investigate which method is more reliable for ranking footwear by using another human-centred test that is based on walking on a level ice surface to more closely reflect real-world conditions with a subset of two types of footwear.

## 2. Materials and Methods

### 2.1. Phase 1: Evaluation of the Mechanical Method

**Figure 1:**
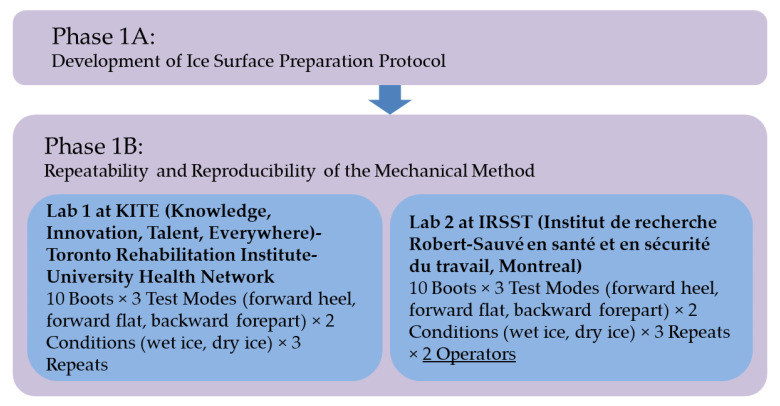
An overview of our testing process used in Phase 1.

Figure 1 shows an overview of our testing process used in this phase.

#### 2.1.1. Phase 1A: Development of an Ice Surface Preparing Protocol for the Mechanical Method

**Figure 2:**
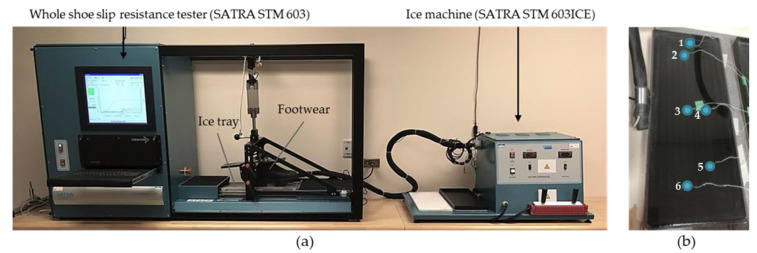
(**a**) The SATRA STM 603 Slip resistance tester (SATRA Technology Centre, Kettering, UK) and the STM 603ICE (SATRA Technology Centre, Kettering, UK) used in the mechanical method to evaluate the coefficient of friction (COF) between footwear and ice surfaces, (**b**) The blue circles show the thermistors positioned under and on the ice surface of the ice machine at KITE (Knowledge, Innovation, Talent, Everywhere)-Toronto Rehabilitation Institute-University Health Network.

One SATRA STM 603 Slip resistance tester and one STM 603ICE were used in both labs (KITE and IRSST) to generate mechanical test results, as shown in Figure 2a. The tests were conducted in ambient temperatures of 23 °C and 21 °C, with relative humidity of 33% and 32–58% at the KITE and IRSST labs, respectively. Frost accumulation was observed when humidity increased; therefore, the frost was wiped away with a wet cloth about three minutes before the measurements. SATRA TM144-2011 specified that the ice temperature setpoint should be −7 °C to form a dry ice surface. However, to be consistent with WinterLab’s ice condition where the dry and wet ice temperatures are −5.5 °C and −1.5 °C, respectively, different setpoint temperatures of the STM 603ICE were tried at both labs. Six thermistors (model SC50F103VN, Amphenol Thermometrics, Inc., St. Marys, PA, USA, for the IRSST lab; Mon-a-therm™ temperature probe, Nellcor Puritan Bennett Inc., Pleasanton, CA, USA, for the KITE lab) were used to measure the actual ice temperature in both labs. Thermistors were placed at different locations, as shown in Figure 2b, three thermistors under the surface of the ice directly on the ice tray metallic surface, and three others on the ice surface that were set in position by a few drops of water that froze in less than a minute. The thermistors were connected to a data logger (USB-6002, National Instruments, TX, USA, for the IRSST lab; Smartreader 8+, ACR Systems, Canada, for the KITE lab). The data logger was then connected to a computer to provide real-time temperature data. The actual ice temperatures were compared to the temperature displayed by the STM 603ICE in order to determine whether the two icy trays produced similar ice conditions when the setpoints were the same.

#### 2.1.2. Phase 1B: Evaluation of the Repeatability and Reproducibility of the Mechanical Method

**Figure 3:**
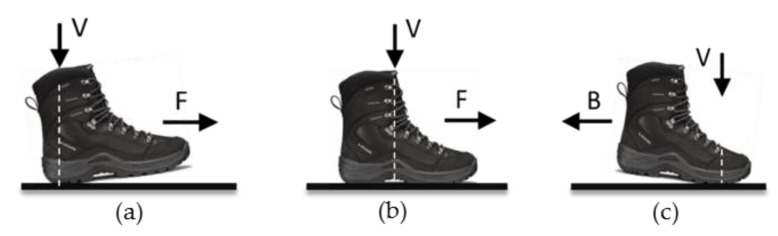
Three test modes: (**a**) Forward heel slip, (**b**) Forward flat slip and (**c**) Backward toe slip, as described in standard mechanical testing (ASTM) F2913-19 [39]. The vertical arrow (V) represents the line of action of the vertical force with respect to the sole-surface contact area. The horizontal arrow represents the forward (F) or backward (B) sliding direction of the footwear relative to the surface.

The inter-laboratory reproducibility was evaluated by comparing results from the two labs using the same protocol. The mechanical test parameters specified by the standard test method ASTM F2913-19 [36] were used to measure the COFs of footwear on ice surfaces. As per this method, a vertical force of 500 N (for Men’s) or 400 N (for Women’s) was applied to the test footwear against the ice test surface. The test surface was then moved horizontally relative to the footwear at 0.3 m/s. The SATRA STM 603 calculated the COF value as a function of time by computing the ratio: horizontal force/vertical force. A series of 5 to 10 successive runs was performed and the final COF was the average of the last 5 consecutive runs, generally showing a variation of less than 10%. The position of the footwear and the line of action of the vertical force with respect to the sole-surface contact area were set to obtain three test modes: forward heel slip, forward flat slip, and backward toe slip, as shown in Figure 3 The tests were conducted on two different ice conditions: dry and wet.

**Figure 4:**
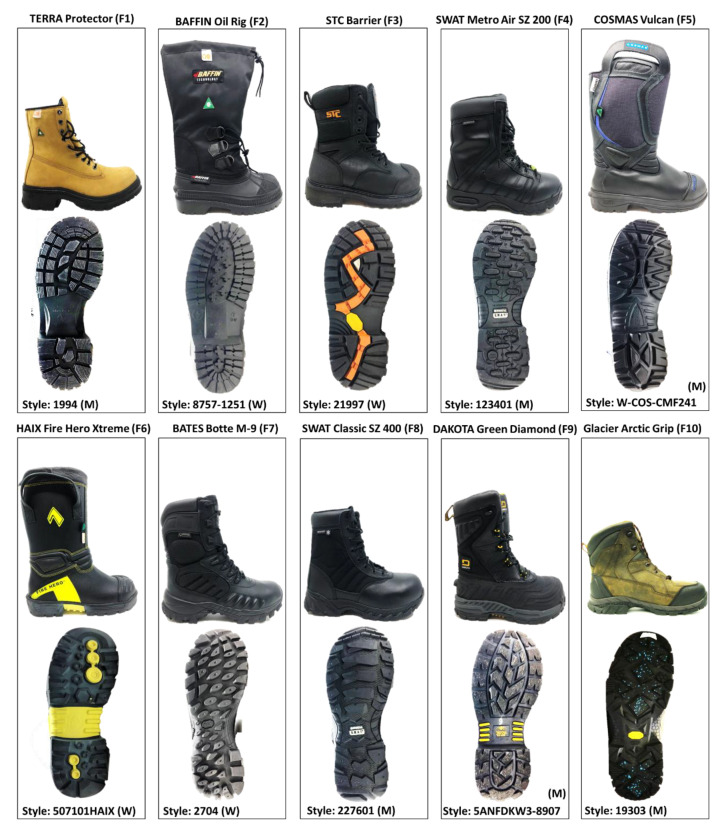
The ten types (F1–F10) of winter footwear tested in this study.

Ten types of occupational footwear (6 men’s and 4 women’s styles) shown in Figure 4 were used. Eight of these boots (F1–F8 in Figure 4) were selected with the help of workplace representatives as they are used daily outdoors in a variety of workplaces, including police departments (F4, F7, F8 in Figure 4), firefighting departments (F5, F6) and municipal services (F1, F2, F3), since they are exposed to slip hazards on ice [40,41]. Two other models (F9 and F10) were added to this set for comparison, as they both included composite materials in their outsoles that have demonstrated excellent slip resistance in previous work [20,21,26]. Boot F9 (Dakota) incorporated Green Diamond technology, which adds metallic particles and other grit to the outsole to create a rough surface. Boot F10 (Wolverine) included Arctic Grip technology, which incorporates microscopic glass fibres oriented vertically to penetrate the ice surface during walking. The left boot was tested in all measurements.

There was one operator at KITE and two operators at IRSST. Each operator ran the test three times for all footwear on dry and wet ice conditions. The goal was to measure repeatability, inter-lab reproducibility and inter-operator reproducibility. For repeatability analysis, two parameters were used: the pooled standard deviation (SD_pooled) [42] and the pooled relative standard deviation, expressed as a percentage ($SD_{relative}\_pooled)$ [43].

(1) $$\bar{COF}=\frac{1}{n}\sum_{j=1}^{n}COF_{j}$$

(2) $$SD=\sqrt{\frac{\sum_{j=1}^{n}{\left(COF_{j}-\bar{COF}\right)}^{2}}{n-1}}$$

(3) $$SD\_pooled_{i}=\sqrt{\frac{\sum_{k=1}^{K}\left(n_{k}-1\right)SD_{k}^{2}}{\sum_{k=1}^{K}\left(n_{k}-1\right)}}$$

(4) $$SD_{relative}\_pooled_{i}=\sqrt{\frac{\sum_{k=1}^{K}\left(n_{k}-1\right)SD_{k}^{2}\;{\bar{COF}}_{k}^{-2}}{\sum_{k=1}^{K}\left(n_{k}-1\right)}}*100$$

where n is the number of values in a series of measurements, the suffice k refers to the different series of measurements and K indicates the total number of series of measurements. The suffice i refers to the ice condition (0 for dry ice and 1 for wet ice). For the repeatability analysis, n=3 and K=30 (each operator having performed 30 tests for each ice surface, i.e., 10 boots × 3 modes).

The inter-operator reproducibility and the inter-laboratory reproducibility were assessed by different analyses of variance (ANOVA). The intent was to identify what the significant effects were, such as operators, and the effect sizes of different independent variables, especially the laboratory effect. Post hoc Tukey multiple comparison tests were also run to determine the significant differences between boot COFs. The ANOVA and Tukey statistical analyses were performed using SPSS statistical software (IBM SPSS Statistics, version 23, IBM, Richmond, VA, USA) [44]. The analyses had a significance level of 0.05. The assumptions underlying the use of the models (homogeneity and normality of the residuals) were verified by examining the model’s standardized residuals. The inter-laboratory reproducibility was also assessed using Bland–Altman (B + A) analyses [45] to gauge the agreement between the measurements taken in the two labs and the coefficient of correlation (R) to estimate the linear correlation between the two series of measurements. These analyses were performed using the overall mean of the COFs from the modes and repeats for each lab, each ice condition and each footwear (COF^mean^).

### 2.2. Phase 2: Comparison with the MAA Human-Centred Method

The MAA test protocol was conducted in WinterLab at KITE. This self-contained lab can be tilted up to 15° as participants walk up and down slopes to test footwear. Eight healthy young adults (range from 20 to 65 years of age) were recruited for testing. Only participants who self-reported that they normally walked outdoors in winter conditions and were not taking any medications or drugs that might affect their ability to balance or walk were recruited. As participants may have adverse medical effects (particularly thrombolytic, ischemic or respiratory events, and/or compromised immunity), due to the cold exposure if they had predisposing conditions (as in high blood pressure, hypercholesterolemia, heart conditions, breathing difficulties or other chronic conditions), participants were asked to decline participation if they had been diagnosed with any of these conditions, or if they had any concerns related to their health as a result of cold exposure. Four female participants were recruited to walk with women’s footwear (F2, F3, F6 and F7,), and four male participants were recruited to test men’s footwear (F1, F4, F5, F8, F9 and F10) in random order. They ranged in age from 20 to 65 years and had no known musculoskeletal dysfunctions or mobility limitations. The demographic information of each participant is shown in Table 1.

The MAA test protocol was approved by the University Health Network Research Ethics Board. Participants provided consent before participating in the study. For safety, participants wore a fall arrest harness and were instructed to use a rope handrail only when they felt unsafe.

Two ice conditions were created in WinterLab: dry cold ice and wet melting ice. For the dry cold ice, the entire WinterLab floor surface was flooded with water and cooled to a temperature of −5.5 ± 1.0 °C. Ice temperature was controlled using tubes circulating glycol coolant along the floor surface, and an ambient air temperature of 2.5 ± 2.0 °C was maintained throughout the experiment. In combination, these factors allowed a smooth, dull, ice walkway surface to be formed with minimal melting at the interface (i.e., with no water visible on the ice surface). The relative humidity in the WinterLab for dry ice conditions was about 45%. Starting with a dry ice surface, wet ice conditions were created by holding the ambient air temperature of the room at 8.0 ± 2.0 °C and the ice surface temperature at −1.5 ± 1.0 °C. The warmer ambient temperatures, in combination with the near-freezing ice temperature, helped to maintain a thin layer of water over the ice surface. The relative humidity in the WinterLab for wet ice conditions was about 36%.

In the current protocol, the slope angle of the walkway is progressively increased by 2° until the first failure. An angle was considered the failure angle if the participant could not initiate gait or if both feet slipped simultaneously while traversing the slope. An angle was deemed as a maximum achievable angle (MAA) if the participant walked successfully on two out of three trials at this angle, and the participant failed on two out of three trials at the angle 1° higher than this angle. A trained observer inside WinterLab detected any slips that the participant encountered during their trials.

For every footwear style (Figure 4), 4 participants walked in 2 directions j (uphill and downhill) under 2 different ice conditions i (wet and dry ice), producing 16 MAAs per footwear. The analysis was done separately for each ice condition. In order to determine an MAA score for each footwear style, two ways of calculating the final MAA score were explored to determine which method would be the most appropriate for sharing with consumers:

1. Mean MAA score: uses the mean value of 8 MAAs, i.e., 4 participants × 2 directions, which represents the performance in-between an ascent and descent on each ice surface (i) as follows:
  (5) $$Mean\;MAA=\;MAA_{i}^{mean}=\;\frac{1}{8}\overset{1}{\underset{j=0}{\sum }}\overset{4}{\underset{k=1}{\sum }}MAA_{ijk}$$
2. where i=0 represents dry ice and 1 for wet ice, j=0 for uphill and 1 for downhill slopes, and k represents the participant number. Minimum MAA score: uses the minimum value of 8 MAAs, i.e., 4 participants × 2 directions, which represents the performance on the more slippery slope of the two directions on each ice surface (i) as follows:
  (6) $$Min\;MAA=\;MAA_{i}^{min}=\;\underset{\begin{array}{c} k=1:4\\ j=0:1 \end{array}}{\min}MAA_{ijk}$$

The angle values from the MAA tests are converted to COF values by taking the tangent of the angles in Equation (7).

(7) MAA_COF = tan(MAA)

The mechanical test results for each footwear style on each ice surface were calculated in two ways: the mean of the COFs from the three modes (flat, heel and forepart) and all repeats as a “Mean values” scenario (COF^mean^) to be compared with $MAA\_COF^{mean}$, and the minimal of the COFs from the three modes (flat, heel and forepart) and all repeats as a “Minimum values” scenario (COF^min^) to be compared with $MAA\_COF^{min}$.

Two parameters, correlation coefficient (R), and residual sum of squared (RSS) are considered to compare mechanical test results at KITE (KITE_COF) and MAA_COF values. The RSS is obtained as follows:

(8) $$RSS=\;\overset{10}{\underset{f=1}{\sum }}{\left(MAA\_COF_{f}-KITE\_COF_{f}\right)}^{2}$$

where f is the footwear number.

Bland–Altman (B + A) analyses [45] were made to evaluate the agreement between the measurements obtained with the mechanical test method from the KITE lab and the measurements obtained using the human-centred MAA method. The comparison was also assessed by the linear correlation coefficient (R) between the measurement series. To facilitate the interpretation and analysis of boot performance; the COFs obtained were compared with the threshold of 0.12, which corresponds to a minimum MAA score of 7° according to the ranking system determined by KITE for the MAA method [25]. If footwear does not achieve a minimum MAA score of 7°, this footwear will not be considered safe to use on icy surfaces. This ranking system was based on the maximum allowable slope for a curb ramp as specified in accessibility guidelines for the built environment [46,47], with the expectation that footwear should prevent slips on commonly encountered icy curb ramps.

### 2.3. Phase 3: Level Walking Test Method for Footwear with Conflicting Rankings

**Figure 5:**
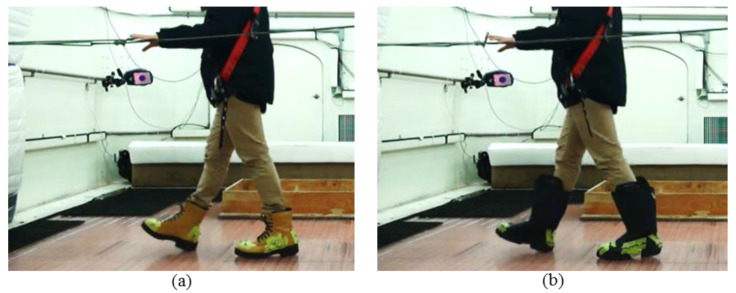
Participant walking in the WinterLab with (**a**) F1 and (**b**) F5.

**Figure 6:**
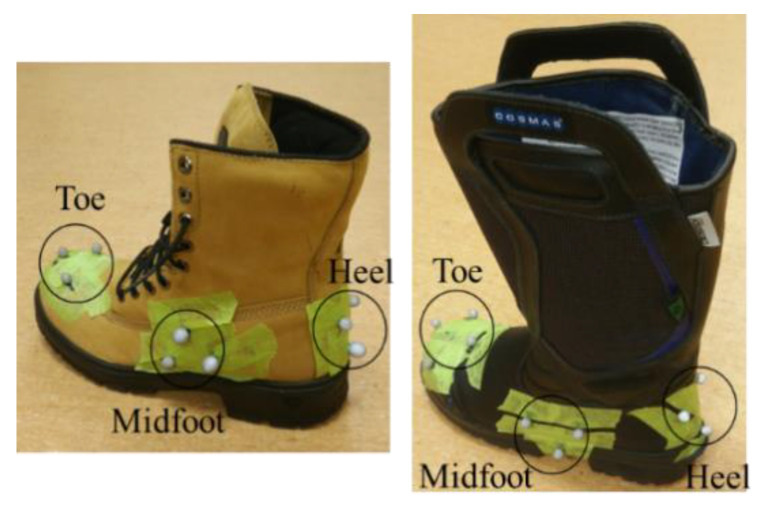
Motion capture markers attached to the F1 (**left**) and F5 (**right**).

Results from Phase 2 showed that for some winter boots, the ratings from the MAA and the mechanical tests conflict. Therefore, a second human-centred test (level walking) protocol was developed to check the number of slips that participants would experience while walking on a level icy surface in WinterLab. For this test, five healthy male participants were recruited. The demographic information of each participant is shown in Table 2. They ranged in age from 20 to 65 years and had no known musculoskeletal dysfunctions or mobility limitations. Participants were instructed to walk back and forth along a linear path to a cadence of 90 beats per minute using an auditory metronome for five minutes. The ninety beats per minute cadence was chosen experimentally because pilot testing demonstrated that this cadence increased the demands on participants and induced a number of slips for most participants. This increased the probability of slipping and enabled a clearer comparison between the two boots. For safety, participants wore a fall arrest harness and were instructed to run one of their hands along a rope handrail shown in Figure 5. A passive motion tracking system comprising 14 Raptor-E and 1 Kestrel cameras by motion analysis recorded the position of markers attached to the footwear. Markers were placed at the toe, midfoot and heel area of the footwear represented in Figure 5 and Figure 6. These marker placements were selected based on previous work [48,49]. The signals were collected at 150 Hz and passed through a Butterworth filter (4th order, zero-phase, 12 Hz cut-off frequency). The participants were asked to walk with their normal gate pattern and there was no control on the stride length.

A previously developed machine learning algorithm [48,49] was used to count the number of slips after extracting the steps by detecting the heel-contact and toe-off from the velocity signal. Slips at the heel and toe were counted. According to a previous study, slips at the heel were defined as a distance the foot travelled beyond 3 cm after heel-contact [49]. In addition, a fall was likely to happen on heel slips exceeding a distance of 10 cm [49]. Therefore, in this paper, 3 cm was adopted as the threshold for counting heel slips (slip distance > 3 cm) and 10 cm as the threshold for hazardous slips (slip distance > 3 cm). However, to the best of our knowledge, there are no previous studies that considered slip distance for the toe slips. Therefore, we counted all toe slips (slip distance > 0 cm).

## 3. Results

### 3.1. Phase 1: Evaluation of the Mechanical Method

#### 3.1.1. Phase 1A: Development of an Ice Surface Preparing Protocol for the Mechanical Method

Monitoring the ice tray’s ice temperatures with thermistors at IRSST and KITE laboratories revealed that the actual ice surface temperature was colder than the setpoint temperature and colder than the temperature reading on the display of the STM 603ICE.

**Figure 7:**
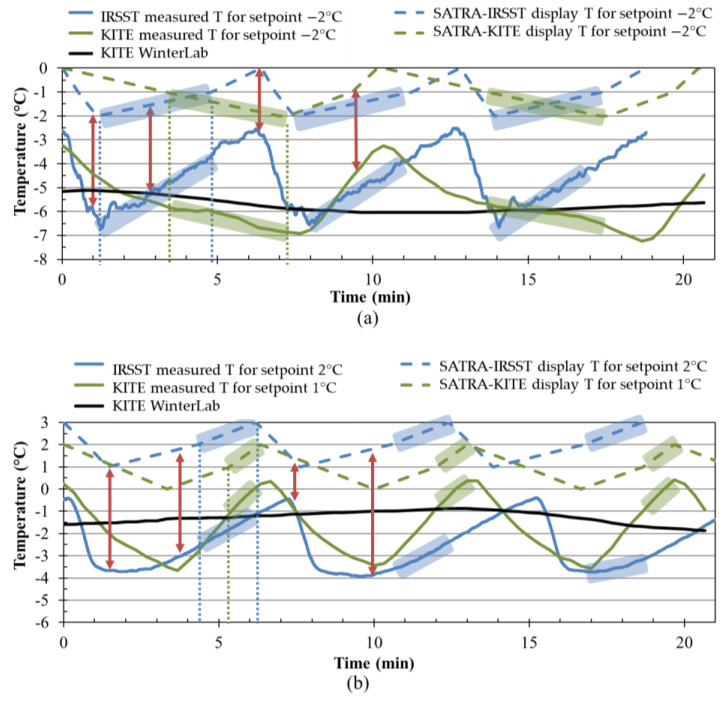
The temperature data recorded from WinterLab and SATRA machines in (**a**) dry ice and (**b**) wet ice conditions for both KITE and *Institut de recherche Robert-Sauvé en santé et en sécurité du travail* (IRSST) laboratories.

Figure 7 shows the ice temperature data obtained from the STM 603ICE ice trays and WinterLab. The dashed blue and green lines are the temperatures displayed on the STM 603ICE, and the solid blue and green lines are the ice temperatures measured by a thermistor attached directly to the ice surface at IRSST and KITE, respectively. The solid black line shows the temperature profile for the ice in WinterLab.

Monitoring of the ice tray’s ice temperatures with thermistors also revealed that the ice surface temperature fluctuates as a function of the ice refrigeration cycle that corresponded to the stop-and-start cycle of the compressor allowing the STM 603ICE to maintain the setpoint temperature. This fluctuation showed slightly different patterns between the two labs: at KITE, it seemed that the ice took less time to warm up than it took to cool, whereas at IRSST it was the reverse, with the ice warming up more slowly but cooling more quickly (Figure 7).

Specific temperature setpoints and restricted temperature ranges for testing on dry and wet ice surfaces were determined for each lab to ensure the ice temperatures measured by the thermistors were as similar as possible at the two labs and matched the WinterLab’s ice temperatures. The temperature setpoint −2 °C was found to be the best for producing a dry ice surface. At this setpoint, the display of the STM603ICE showed the temperature fluctuated between 0 °C and −2 °C, which corresponds to a fluctuation of the actual temperature of the ice between −2.5 °C and −6.5 °C at IRSST, and between −3 °C and −7 °C at KITE. In order to be as close as possible to the WinterLab’s dry ice condition, the COF values were measured when the displayed temperature was in the range of (−1 °C, −2 °C) at KITE (highlighted in green in Figure 7a) and (−2 °C, −1 °C) at IRSST (highlighted in blue in Figure 7a). The test started when the displayed temperature read −1 °C at KITE (−2 °C at IRSST) and stopped when the displayed temperature read −2 °C at KITE (−1 °C at IRSST). Although the COFs from the 5 consecutive runs could vary in opposite directions in the two labs, the final COF was the average of the last 5 consecutive runs. Therefore, the direction of ice temperature changes should not affect the final COF. WinterLab’s wet ice condition (−1.5 °C) was most closely replicated when the temperature setpoint was 1 °C and the displayed temperature was in the range of (1 °C, 2 °C) at KITE (2 °C setpoint and (2 °C, 3 °C) range at IRSST). As shown in Figure 7b, the test started when the displayed temperature read 1 °C (2 °C at IRSST) and stopped when the displayed temperature read 2 °C at KITE (3 °C at IRSST). The time slot to carry out the test was about 1 to 3 min depending on the ice condition and lab. This duration was enough for one test, i.e., a series of 5 to 10 successive runs, which took less than a minute.

#### 3.1.2. Phase 1B: Evaluation of the Repeatability and Reproducibility of the Mechanical Method

The results show that for dry ice, the SDs varied between 0.000 and 0.040 depending on conditions; the $SD\_pooled_{0}$ was 0.014, and the $SD_{relative}\_pooled_{0}$ was 9.0%. For wet ice, SDs varied between 0.000 and 0.020, the $SD\_pooled_{1}$ was 0.009, and the $SD_{relative}\_pooled_{1}$ was 16.8%. Overall, the intra-operator repeatability for dry ice generally produced lower relative SDs than for wet ice. Therefore, a small deviation from the average value implies acceptable repeatability of the mechanical results over similar conditions.

The inter-operator reproducibility was verified using two operators’ readings from IRSST. The results from ANCOVA show that the measurements from two different operators at IRSST are similar (p > 0.05) and, therefore, the factor “operator” has no effect on the COF values. It is also worth mentioning that the covariates temperature and relative humidity did not have a significant effect (p > 0.05) on the measured COFs within the temperature and relative humidity ranges of the IRSST laboratory during the tests.

**Figure 8:**
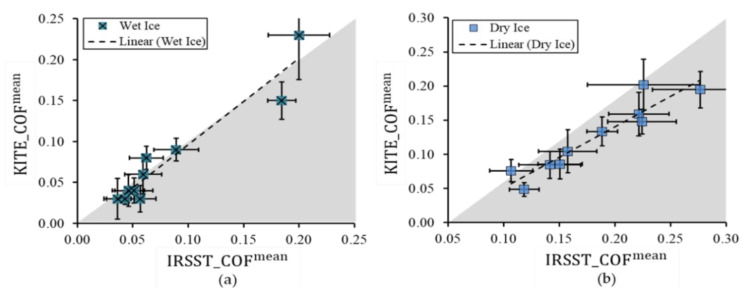
A comparison between mean COFs obtained from SATRA devices in KITE and IRSST for (**a**) wet ice and (**b**) dry ice surfaces. The gray area includes the points where IRSST_COF^mean^ ≥ KITE_COF^mean^.

**Figure 9:**
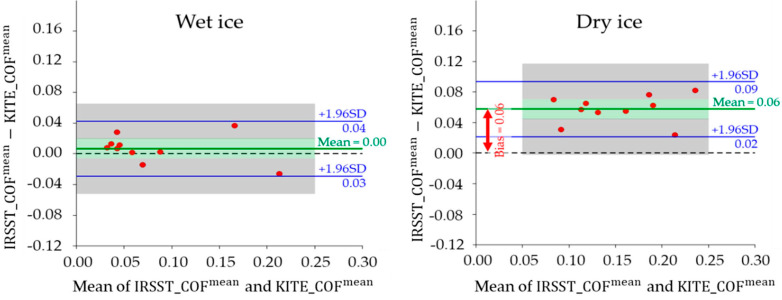
The Bland–Altman plot for COF^mean^ values from KITE and IRSST devices on (**a**) wet and (**b**) dry ice. The green and gray zones represent the confidence intervals around the mean and the LoA, respectively.

**Figure 10:**
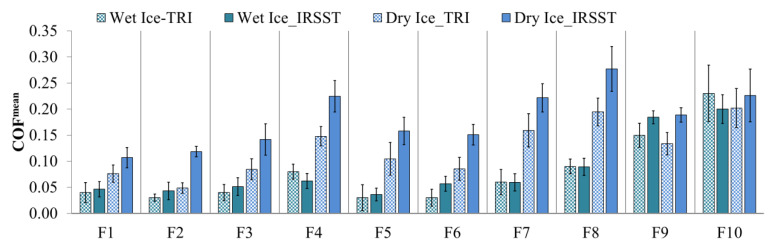
The mean COF values from KITE and IRSST mechanical test on wet and dry ice surfaces.

The outcomes from two sites, KITE and IRSST, are significantly different (p < 0.001), especially in the dry ice condition with a higher effect size ($\eta_{p}^{2}=0.76)$ compared to wet ice ($\eta_{p}^{2}=0.07$). This means that the two labs produced different COFs; however, the differences were more noticeable for measurements on dry ice than on wet ice. This rejects the inter-lab reproducibility in both ice conditions, which might be due to differences in the refrigeration cycles (see Figure 7a), the lab’s environmental parameters and the features of the ice trays in the two labs, e.g., the warmer and colder areas were not distributed similarly on the two trays. Therefore, a perfect match between the procedures at KITE and IRSST would be desirable. Figure 8 shows the mean COFs on wet and dry ice conditions. The dashed black lines show the linear trends of the COF results. The high correlation coefficients (R=0.96 and R=0.94 for wet and dry ice respectively) indicate the high linear relation between the two values. Figure 9 shows the small limits of agreement (LoA = 0.036) in Bland–Altman (B + A) analysis. Another observation is that the SATRA device at KITE always reported lower COFs than the IRSST SATRA in the dry ice condition. The average systematic bias between the two values was about 0.06, which is shown in Figure 9. This bias is about zero in the wet ice condition. Figure 10 depicts the mean COF values for IRSST and KITE on wet and dry ice for all footwear. The IRSST and KITE both agreed on the two best slip-resistant footwear in wet ice conditions. The F10 footwear had the best slip-resistance performance with maximum mean COFs on wet ice, and the second-best was the F9. However, the results differed in the dry ice condition.

### 3.2. Phase 2: Comparison with the MAA Human-Centred Method

**Figure 11:**
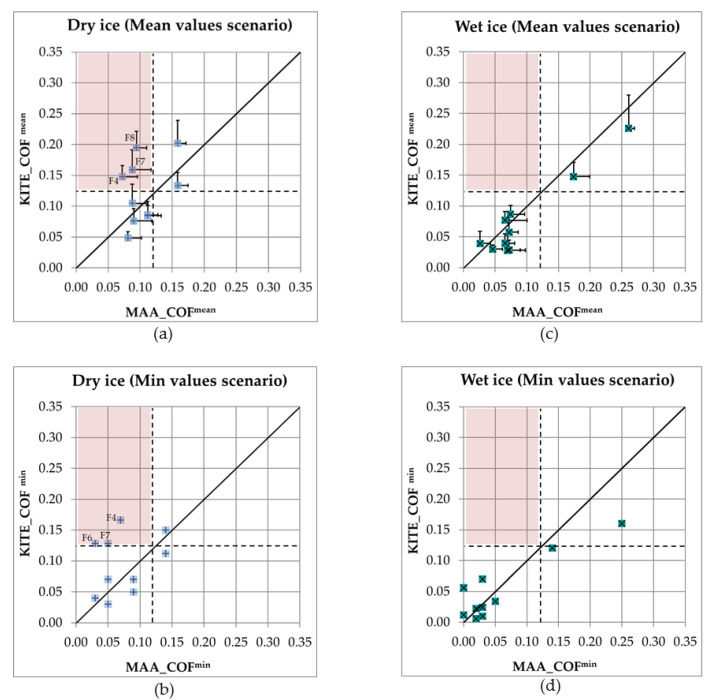
A comparison between COFs obtained from the mechanical method at KITE and the human-centred MAA method for (**a**) Mean values scenario on dry ice, (**b**) Minimum values scenario on dry ice, (**c**) Mean values scenario on wet ice, and (**d**) Minimum values scenario on wet ice. The solid black line represents what would be a perfect agreement where MAA_COF^mean^ = KITE_COF^mean^ and MAA_COF^min^ = KITE_COF^min^. The black dash lines represent the set threshold of 0.12. The red zone represents the area where the mechanical method incorrectly classifies the footwear as one snowflake footwear, while the MAA method ranked footwear in that zone as unsafe footwear for icy surfaces.

In this phase, the results of all 10 boots were compared. Table 3 presents the COF values for all footwear obtained from different methods (mechanical from both labs and MAA), in mean and minimum values scenarios, for dry and wet ice conditions. Figure 11 presents the results obtained from the mechanical method at KITE compared to the results obtained from MAA. For dry ice (Figure 11a,b), substantial differences are seen between the results of the two methods. With the MAA method, only boots F9 and F10 had high COFs, exceeding the threshold of 0.12 (Table 3). With the mechanical method, on the other hand, several other boots also had high COFs, not only exceeding the threshold of 0.12, but also revealing some performance results inconsistent with those assessed with the MAA method. For example, boots F4, F7 and F8 showed good slip resistance quality when tested by the mechanical method.

However, these results disagreed with the MAA method. For wet ice (Figure 11c,d), moderate differences are seen between the results of the two methods. They both show better performances for boots F9 and F10, which outperform the other boots. This is observed for both mean MAA and minimum MAA scores. However, some boots showed different performances depending on the testing method. For example, with the mean values scenario on wet ice (Table 3), the results from the mechanical tests indicated that F1 and F5 were not different in terms of the slip resistance (COF^mean^ = 0.04 and 0.03, respectively at KITE). Mean MAA_COF, on the other hand, showed that the two boots had different slip-resistance qualities (MAA_COF^mean^ = 0.03 and 0.07, i.e., angle of 1.7° vs. 4.0°, respectively) highlighted in Table 3. This table also shows that the F10 and F9 footwear styles achieve significantly higher COF compared to the other 8 footwear styles in both ice conditions. The Arctic Grip technology in F10 performs better on wet ice conditions than dry ice; however, the slip resistance quality of the Green Diamond technology in F9 remains consistent between two different types of ice conditions. Overall, the results demonstrate that the wet ice condition seems to be more challenging for the other 8 footwear styles, where the majority achieved lower values compared to the dry ice.

**Figure 12:**
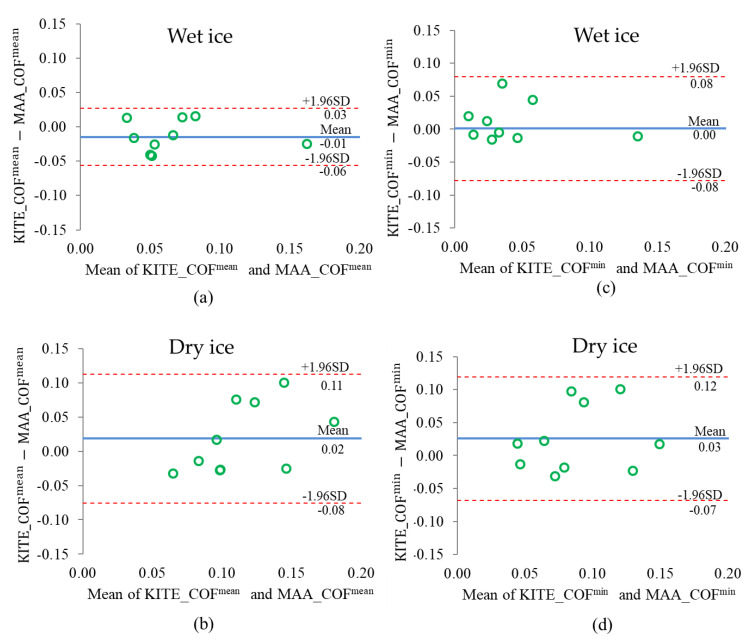
B&A plots for Mean values scenario on (**a**) wet and (**b**) dry ice, and Minimum values scenario on (**c**) wet and (**d**) dry ice conditions.

Using the mean values scenario on the wet ice condition, the residual sum of squared (RSS) was 0.01, which shows a small deviation between KITE_COF^mean^ and MAA_COF^mean^, and the correlation coefficient was 0.95 (Table 4). On the dry ice, there was an RSS of 0.03 and a weak correlation (R = 0.34) between KITE_COF^mean^ and MAA_COF^mean^ using the mean values scenario. The B + A analysis (Figure 12) confirmed that the two methods were not in agreement for dry ice, with a high LoA of 0.12 (bias = 0.02), but in better agreement on wet ice, with a low LoA of 0.07 (bias = −0.01).

### 3.3. Phase 3: Level Walking Test Method for Footwear with Conflicting Rankings

The results from Phase 2 indicated that the mechanical and MAA methods ranked the performance of the footwear differently. For example, when considering MAA mean scores on the wet ice condition, F5 had a higher MAA score compared to F1 (0.07 vs. 0.03, as shown in Table 3). The mechanical test results from both labs indicated that the two boots were very similar in terms of the slip resistance (0.03 vs. 0.04 in KITE and 0.04 vs. 0.05 in IRSST). In addition, the post hoc Tukey multiple comparison tests with the mechanical method from both labs grouped F1 and F5 into the same homogeneous subsets (subsets of means that do not differ from each other at p < 0.05). However, the same test on MAA values groups F1 and F5 in different subsets, which means that they are significantly different from each other at p < 0.05. Therefore, the test proposed in Phase 3 is used to count the: number of heel slips, toe slips, and hazardous slips (slip distance > 10 cm) in order to resolve these conflicting results.

**Figure 13:**
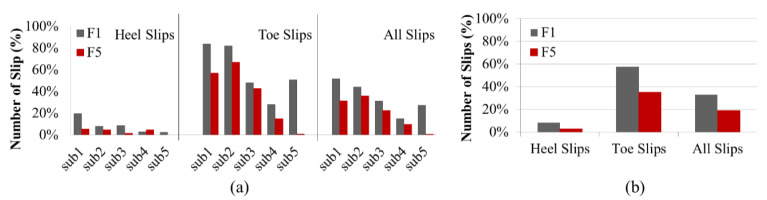
(**a**) The number of slips per participant with F1 and F5, (**b**) Total number of slips from all participants with F1 and F5.

**Figure 14:**
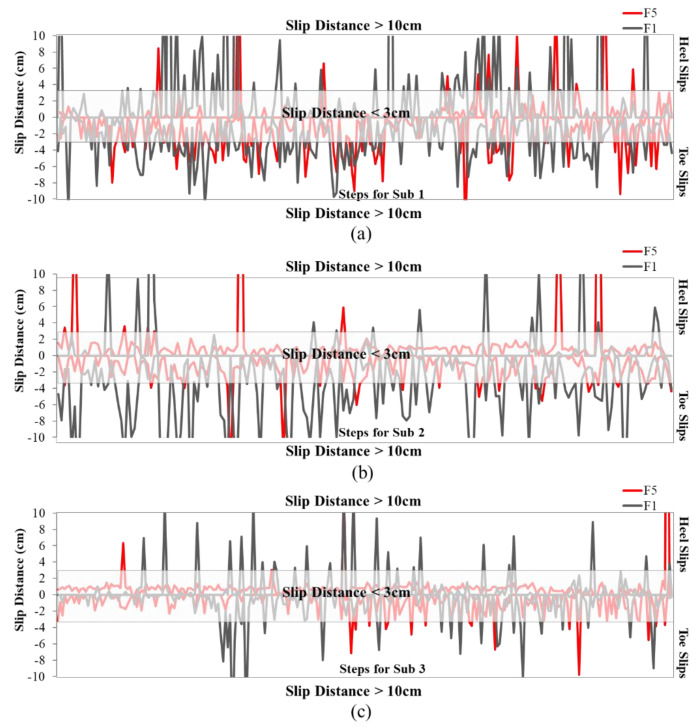

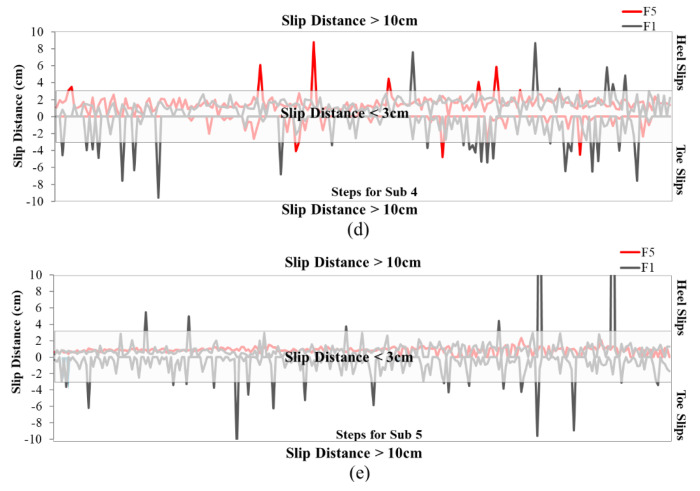
The slips distance for both heel and toe slips across all steps taken by (**a**) Sub 1, (**b**) Sub2, (**c**) Sub 3, (**d**) Sub 4, and (**e**) Sub 5.

Figure 13a showed that all participants except one (Sub4 with 3% vs. 5% slips) experienced more toe and heel slips with F1 than with F5. Moreover, as depicted in Figure 13b, the total number of both heel and toe slips was higher for F1 compared to F5. This figure also indicated that F1 had about twice the number of slips as F5 (33% vs. 19%, respectively), considering all participants and all types of slips. The large standard deviation in the number of slips, for example ranging from 3% to 20% for heel slips, was likely due to different gait patterns among participants. These differences in the gait patterns were also highlighted in Figure 14. This figure showed the region where the slip distance was greater than 3 cm and 10 cm for heel slips for each participant. For instance, Figure 14a,b show that both Sub1 and Sub2 slipped more than other participants. In addition, Figure 14d demonstrated that Sub4 had no hazardous slips (>10 cm) during walking on the wet ice in WinterLab. The analyses showed that F1 had twice the number of hazardous heel slips as F5 (25 vs. 10 occurrences). All participants experienced more hazardous slips with F1 than with F5. The results indicated that F1 was less slip-resistant than F5, which was consistent with the MAA results, but not with the mechanical test results.

## 4. Discussion

### 4.1. Phase 1: Evaluation of the Mechanical Method

The intra-operator repeatability of the mechanical method used in this study was generally acceptable for all operators (IRSST and KITE), with pooled relative SDs of 9.0% for dry ice and 16.8% for wet ice. The inter-operator reproducibility (IRSST) was satisfactory since the trained operator did not have any significant effect on the measurements. In a shoe-on-quarry tile inter-laboratory study of the standardized test method ASTM F2913-19 involving 10 labs with one single model of shoe, the repeatability SD for the heel slip mode was 0.020 (relative SD of 3.9%) on a wet quarry tile and 0.044 (4.6%) on a dry quarry tile, as well as a reproducibility SD of 0.046 (relative SD of 9.0%) on a wet quarry tile and 0.086 (8.9%) on a dry quarry tile [50]. It should be noted that the mean COF of the boot tested (0.513 on a wet quarry tile and 0.964 on a dry quarry tile) in the previous study was higher than those recorded in this study on ice (COF < 0.277), given that the quarry tiles are less slippery than ice surfaces. In order to improve the precision of the mechanical method, it would be useful to minimize the sources of variability and to improve the mechanical testing procedures. The COFs measured were low in some cases, especially on the wet ice surface. A higher resolution of the data acquisition system of the STM 603 device, which is currently 0.01, would be preferable. We note that the inter- and intra-operator SDs are in the same order as our instrument resolution for the STM 603 device, which is 0.01. This means that our ability to distinguish between the performances of two different boots would not change unless we could improve both, and there is limited benefit to improving just one or the other.

Reconsidering the choice of the experimental unit (mean of last 5 consecutive runs) may also be warranted. Although an a posteriori assessment showed that the variation in the last 5 consecutive runs was 10% or less in most, some cases did reveal variations exceeding 10% over successive runs, especially on wet ice. More in-depth study of these variations in successive runs would be valuable, especially as the COF values are heavily influenced by the ice refrigeration cycle, which can show differing rates of change over the cycle. It should be noted that this experimental unit is not suited to soles with crampons or grit, like boot F9 with Green Diamond technology. For that boot, the COF of the first test run would probably have been more representative of the sole slip resistance compared to the mean of the five measurements taken when the grit had gone through the same grooves several times.

Regarding inter-laboratory reproducibility, the analyses showed that the measurements with wet ice were equivalent in both labs with respect to both similar COF values obtained and ranking of the boots (in which boots F9 and F10 stood out as distinctly better performers than the other models). For dry ice, the COF values recorded at the IRSST lab were systematically higher than those obtained at KITE, with a bias of 0.06. Still, the agreement between the two labs was relatively good and the linear correlation was high.

There are a number of sources of variability that may explain the differences noted between the two labs. First, the refrigeration cycles showed different patterns, especially for dry ice. For this type of ice, the test windows had to be defined at a time when the ice was warming up at the IRSST (from −2 °C to −1 °C) and cooling down at the KITE (from −1 °C to −2 °C). The differences in patterns observed in the refrigeration cycles could be due to possibly different operations of the ice trays (among other things, the warmer and colder areas are not located at the same places on the two trays). They may also be due to the laboratory environment, where the temperature, relative humidity and speed of circulation of the ambient air can affect thermal exchange between the air and the ice surface and have an impact on the ice refrigeration cycle.

Another source of variability can be found in the procedure for removing frost from the ice surface with a wet cloth. While meetings were held with operators from the two labs to standardize the procedure and use the same cloth, a better match between the procedures would be desirable (amount of water used, length of time that elapses between the use of the wet cloth and the test run). In future, the impacts of these variables on the results will be investigated by conducting separate studies; we will also include standard methods to measure the ice roughness to better quantify the ice surface morphology.

### 4.2. Phase 2: Comparison with the MAA Human-Centred Method

The analysis of the data indicated that the mechanical test results were not in agreement with the MAA test results on dry ice. Especially, the MAA method found boots F4, F7 and F8 as among the lowest performers (below 0.12), while the mechanical method rated them as being among the best performers. It may therefore be possible that the mechanical method overestimates the performance of some boots in certain cases. However, it is worth noting that both methods found that boots F9 and F10 offered good slip resistance. There were several reasons that could explain these variabilities. Firstly, the test parameters used in the mechanical method did not reflect the differences in human gait patterns, as is the case with several mechanical methods [51,52]. Secondly, the ambient temperature and humidity were not tightly controlled in the KITE and IRSST labs, which might change the dry ice characteristics.

The dry ice condition was defined by the absence of any water film visible on the surface. However, we note that determining that there is no water film on the ice surface can be challenging, and therefore, the dry ice condition should be more carefully defined [53]. The mechanical method would have to be improved by changing some of the test parameters, such as vertical force, slipping speed and heel contact angle, as suggested by previous studies [34,35,36] to be comparable with the MAA results.

On the other hand, the mechanical test and MAA test results seemed to be in agreement in wet ice conditions. The COF values on wet ice surfaces were generally very low, except for F9 and F10. As shown in Figure 11c,d, there was a cluster of data points with low COFs, and two points with high COF. Therefore, the correlation between the two test methods on wet ice surface should be interpreted carefully. More footwear with moderate slip-resistance on wet ice surfaces need to be tested to determine the correlation between the two methods. Furthermore, there are two types of COF: static (SCOF) and dynamic (DCOF). High SCOF helps to prevent slip initiation and high DCOF helps to stop the slip; therefore, both SCOF and DCOF values are important for preventing slip-related falls [16,17]. The mechanical test measures the DCOF and the MAA test measures both SCOF and DCOF. Therefore, the MAA test could be used to evaluate the slip resistance of footwear in preventing slip initiation and in stopping the slip.

In addition, although efforts were made to obtain ice surfaces with the ice tray that were as similar as possible to the ice surfaces of the WinterLab, the temperatures at the ice surface, and especially the ambient air conditions, were still not the same. Moreover, the conditioning of the boots prior to the tests was not done at the same temperatures. Mechanical tests were done at 23 °C ambient temperature, while MAA testing was done at 2.5 °C ambient temperature for dry ice condition and 8.0 °C for wet ice condition. It is, therefore, possible that this parameter has an impact on the difference in the results between the two test methods. The decision not to condition the boots at a colder temperature, for the mechanical method developed in this study, was made because keeping the boot cold is quite a challenge in a laboratory where the ambient air temperature is 23 °C and the temperature of the boot sole is increasing at a rate of approximately 1 °C per minute from 0 °C to 10 °C, and because conditioning the footwear to the colder temperatures would not allow running as many repetitions in one day as conditioning at the laboratory ambient temperature. For the further development of the mechanical method, however, it could be useful to rerun some tests by conditioning the boots in colder temperatures.

The mean value scenario was a reasonable approach to compare the mechanical and MAA results because it took into account different ways of slipping (3 slip modes for the mechanical test, 2 slipping directions in the MAA test) to evaluate the overall performance of the boots. However, the minimum values scenario seems to be the most appropriate when providing information on footwear performance to the consumer, as done by the KITE for the MAA method [25]. One reason is that the minimum MAA gives better protection than the mean MAA when considering that the consumer, while wearing only one pair of boots, can walk in the real world on different ice surfaces, encounter lumps, steps and uneven terrain, and might not be aware of changes between wet and dry ice surfaces resulting, for example, from patches of sun and shade and different times of the day [54]. The other reason is that it seems unreasonable to quote more than the minimum MAA as long as there are still slipping phenomena that are not sufficiently understood. As an example, the ‘clogging effect’ observed during the MAA test occurs shortly after a longer slip on ice with an Arctic Grip kind of material, where a layer of ice scrapings seems to temporarily adhere to the outer surface of the sole and drastically reduces friction. The minimum MAA is a conservative estimate of footwear performance—it can be seen as a worst-case scenario and should therefore be considered from a consumer perspective.

The low number of participants for MAA testing is a limitation of the study. The criteria used in sample size estimation for the MAA method meant that a 95% confidence interval (CI) for the mean maximum achievable angle for each type of footwear should be no more than two degrees in total width. In other words, we determine the sample size and number of repetitions that will yield a 95% CI of x ± 1.0°. An internal study was conducted at KITE in 2014, with 15 male and 15 female participants, to establish the required sample size. The results revealed that a sample size of 4 with a repetition of 1 yielded a 95% CI of x ± 0.95°, and a sample size of 8 with a repetition of 3 yielded a 95% CI of x ± 0.48°. Therefore, a sample size of 4 is considered as a good compromise between cost efficiency and accuracy. Future work will include a study to consider more participants and more types of footwear to estimate the sample size to develop a more accurate model.

Finally, it may be worthwhile to investigate the performance of the boots under real conditions in the field to get a better assessment of the value of the mechanical method in the laboratory and under a variety of real winter conditions.

### 4.3. Phase 3: Level Walking Test Method for Footwear with Conflicting Rankings

The comparison between the two boots, based on the three parameters, indicated that F1 was less slip-resistant than F5. This outcome was consistent with the results from the MAA test method, but not with those from the mechanical test method. The fact that the MAA and level walking tests were done under the same environmental conditions (wet ice inside the WinterLab) was certainly a factor in the similarity of the results. It should be noted, however, that even when walking conditions were different (specific walking pace vs. self-selected pace, level walking back and forth vs. ascending and descending), participants experienced the same performances with the two boots.

Even though there was high variability between participants, the phase 3 test protocol, based on the number of slips and slip distance (instead of COF), showed good potential for footwear slipperiness comparison. However, the detection of slip events and slip distances was very challenging. The small sample size was one of the limitations of this phase. Other limitations include a relatively short walking distance for the testing protocol in both phases 2 and 3, where only young participants were tested. More research will be needed to improve this method. Future work should include a protocol similar to that of phase 3, but taking place outdoors in real-world icy conditions, while the numbers of slip events are tracked by a wearable slip-detector (currently in development). In addition, it may be useful to test other conditions that produced conflicting outcomes, such as on dry ice with boots F1 and F4 (F1 < F4 with the mechanical method, but F1 ≈ F4 with MAA), or boots F8 and F10 (F8 > F10 with the mechanical method at IRSST, but F8 = F10 at KITE, and F8 << F10 with MAA). An improved level-walking test could provide further information on the performance of the footwear, which could be used as an additional method to validate the MAA test method. In future, we will investigate different factors such as participants’ demographic, walking speed, and step length in this proposed human-centred protocol to provide a more rigorous statistical analysis.

## 5. Conclusions

Overall, this work provided a better understanding of the limitations of the mechanical test method using the SATRA ice tray for measuring slip resistance. Our findings indicate that the mechanical method must be further refined to make its results more comparable to human-centred methods, which are more likely to reflect real-world slip resistance performance.

The mechanical slip resistance testing results obtained in phase 1 from our two labs showed a high linear correlation (>0.94) and good agreement on both ice surfaces. However, they revealed a bias (~0.06) between the two labs on the dry ice condition. Comparing these mechanical test results to the human-centred test results in phase 2 showed a better correlation in the wet ice condition (R = 0.95) compared to the dry ice condition (R = 0.34). Finally, the rating of the footwear slip resistance based on the number of slips counted in the alternate human-centred testing methods in phase 3 was consistent with the rating by the human-centred test method (phase 2), but not the mechanical method (phase 1).

## Figures and Tables

**Table 1:** The participants’ demographics (Phase 2).

Subject #	Age	Sex	Height (m)	Weight (kg)	BMI (kg/m^2^)
Sub1	23	F	1.60	60	23.4
Sub2	23	F	1.63	53	20.2
Sub3	25	F	1.68	77	27.4
Sub4	22	F	1.73	67	22.5
Sub5	19	M	1.68	63	22.3
Sub6	28	M	1.73	76	25.5
Sub7	20	M	1.81	58	17.7
Sub8	25	M	1.88	86	24.4

**Table 2:** The participants’ demographics (Phase 3).

Subject #	Age	Height (m)	Weight (kg)	BMI (kg/m^2^)
Sub1	25	1.73	58	19.5
Sub2	22	1.83	80	24.1
Sub3	41	1.68	65	23.0
Sub4	32	1.74	55	18.2
Sub5	20	1.73	68	22.8
Average	28	1.74	65	21.5

**Table 3:** COF values for all footwear obtained from different methods and ice conditions.

	Dry Ice						Wet Ice					
	Mean Values Scenario			Minimum Values Scenario			Mean Values Scenario			Minimum Values Scenario		
Footwear	KITE_COF^mean^	IRSST_COF^mean^	MAA_COF^mean^	KITE_COF^min^	IRSST_COF^min^	MAA_COF^min^	KITE_COF^mean^	IRSST_COF^mean^	MAA_COF^mean^	KITE_COF^min^	IRSST_COF^min^	MAA_COF^min^
F1	0.08	0.11	0.09	0.04	0.07	0.03	0.04	0.05	0.03	0.01	0.02	0.00
F2	0.05	0.12	0.08	0.03	0.09	0.05	0.03	0.04	0.05	0.02	0.02	0.02
F3	0.08	0.14	0.11	0.07	0.10	0.09	0.04	0.05	0.07	0.02	0.03	0.03
F4	0.15	0.22	0.07	0.13	0.18	0.03	0.08	0.06	0.07	0.06	0.03	0.00
F5	0.10	0.16	0.09	0.07	0.11	0.05	0.03	0.04	0.07	0.01	0.02	0.02
F6	0.09	0.15	0.11	0.05	0.12	0.09	0.03	0.06	0.07	0.01	0.03	0.03
F7	0.16	0.22	0.09	0.13	0.17	0.05	0.06	0.06	0.07	0.03	0.03	0.05
F8	0.19	0.28	0.09	0.17	0.22	0.07	0.09	0.09	0.07	0.07	0.06	0.03
F9	0.13	0.19	0.16	0.11	0.18	0.14	0.15	0.18	0.17	0.12	0.16	0.14
F10	0.20	0.23	0.16	0.15	0.18	0.14	0.23	0.20	0.26	0.16	0.15	0.25

Note: the highlighted cells correspond to the two footwear with conflicting results.

**Table 4:** Residual Sum of Squares (RSS) with respect to the line (y = x) and correlation coefficient ‘R’ between KITE_COF and MAA_COF on two different scenarios.

Mean Values Scenario				Minimum Values Scenario			
Dry Ice		Wet Ice		Dry Ice		Wet Ice	
R	RSS	R	RSS	R	RSS	R	RSS
0.34	0.03	0.95	0.01	0.32	0.03	0.90	0.01

## Data Availability

Data sharing not applicable due to ethical and privacy restrictions.
